# Preclinical exploration of combining plasmacytoid and myeloid dendritic cell vaccination with BRAF inhibition

**DOI:** 10.1186/s12967-016-0844-6

**Published:** 2016-04-14

**Authors:** Jurjen Tel, Rutger Koornstra, Nienke de Haas, Vincent van Deutekom, Harm Westdorp, Steve Boudewijns, Nielka van Erp, Stefania Di Blasio, Winald Gerritsen, Carl G. Figdor, I. Jolanda M. de Vries, Stanleyson V. Hato

**Affiliations:** Department of Tumor Immunology, Radboud Institute for Molecular Life Sciences, Radboud University Medical Center, 6500 HB Nijmegen, The Netherlands; Department of Medical Oncology, Radboud University Medical Center, Nijmegen, The Netherlands; Department of Pharmacy, Radboud University Medical Center, Nijmegen, The Netherlands

**Keywords:** Vemurafenib, DC vaccination, Plasmacytoid DCs, BDCA1 + myeloid DCs

## Abstract

**Background:**

Melanoma is the most lethal type of skin cancer and its incidence is progressively increasing. The introductions of immunotherapy and targeted therapies have tremendously improved the treatment of melanoma. Selective inhibition of BRAF by vemurafenib results in objective clinical responses in around 50 % of patients suffering from BRAFV600 mutated melanoma. However, drug resistance often results in hampering long-term tumor control. Alternatively, immunotherapy by vaccination with natural dendritic cells (nDCs) demonstrated long-term tumor control in a proportion of patients. We postulate that the rapid tumor debulking by vemurafenib can synergize the long-term tumor control of nDC vaccination to result in an effective treatment modality in a large proportion of patients. Here, we investigated the feasibility of this combination by analyzing the effect of vemurafenib on the functionality of nDCs.

**Methods:**

Plasmacytoid DCs (pDCs) and myeloid DCs (mDCs) were isolated from PBMCs obtained from buffy coats from healthy volunteers or vemurafenib-treated melanoma patients. Maturation of pDCs, mDCs and immature monocyte-derived DCs was induced by R848 in the presence or absence of vemurafenib and analyzed by FACS. Cytokine production and T cell proliferation induced by mature DCs were analyzed.

**Results:**

Vemurafenib inhibited maturation and cytokine production of highly purified nDCs of healthy volunteers resulting in diminished allogeneic T cell proliferation. This deleterious effect of vemurafenib on nDC functionality was absent when total PBMCs were exposed to vemurafenib. In patients receiving vemurafenib, nDC functionality and T cell allostimulatory capacity were unaffected.

**Conclusion:**

Although vemurafenib inhibited the functionality of purified nDC of healthy volunteers, this effect was not observed when nDCs were matured in the complete PBMC fraction. This might have been caused by increased vemurafenib uptake in absence of other cell types. In accordance, nDCs isolated from patients on active vemurafenib treatment showed no negative effects. In conclusion, our results pave the way for a combinatorial treatment strategy and, we propose that combining vemurafenib with nDC vaccination represent a powerful opportunity that deserves more investigation in the clinic.

**Electronic supplementary material:**

The online version of this article (doi:10.1186/s12967-016-0844-6) contains supplementary material, which is available to authorized users.

## Background

Melanoma is a highly malignant melanocyte-derived tumor with a rising incidence. It is the most lethal of all skin cancers, accounting for 80 % of all skin cancer related deaths while representing only 4 % of all cases [1]. Recently, advances in both targeted therapies, as well as, immunotherapy have finally begun to show effectiveness against metastatic melanoma [2–7]. Mutations in the BRAF signaling pathway have been identified as one of the drivers of melanoma. Mutations in the *BRAF* gene can be detected in 50–60 % of cutaneous melanomas and mutations in the *NRAS* gene in 10–20 % [8, 9]. These mutations lead to constitutive activation of the mitogen activated protein kinase (MAPK) pathway, which in turn, results in increased cell proliferation and survival [10, 11]. Targeted agents directed against the MAPK pathway, mostly small molecule inhibitors, such as vemurafenib, have recently demonstrated clinical efficacy in metastatic disease [2, 3, 12]. Vemurafenib is a selective inhibitor of mutated BRAF and shows great efficacy in metastatic melanoma patients harboring the BRAFV600 mutation [2, 3, 12]. Objective clinical responses are seen in around 50 % of all patients and the drug is generally well tolerated [2, 3, 12]. Despite the rapid responses and high response rates, drug resistance develops in the vast majority of patients, which clearly is an obstacle for achieving long-term tumor control. Even more, it has been reported that tumors might become dependent on vemurafenib, which has led to the postulation of intermitted treatment schedule in order to prevent resistance [13].

The other novel treatment modality for melanoma, which even was designated as breakthrough of the year 2013 [14], is the use of immunotherapy. Broadly immunotherapy can be divided in cellular immunotherapy, such as dendritic cell (DC) vaccination, and antibody-based immunotherapy, such as immune check point blockade [15]. DCs are professional antigen presenting cells that can be loaded with melanoma-associated antigens and used to mount an immune response against tumor cells by activating cytotoxic T cells. DC vaccination for melanoma has been explored in many phase I and II studies. Thus far, virtually all of these clinical studies have been performed with ex vivo differentiated monocyte-derived DCs (moDCs) or from CD34^+^ progenitors. Although numerous vaccination studies demonstrated the immunogenicity of tumor antigen-loaded DCs, the number of objective clinical responses has been limited, hampering its implementation as a novel form of standard treatment. However, DC vaccination still has untapped potential as, some stage IV melanoma patients did experience long lasting clinical responses. Additionally, in stage III melanoma there was a favorable overall survival benefit after adjuvant DC vaccination [16]. Additionally, recent trials exploiting naturally occurring DC subsets (nDCs), which circulate in the blood, as a vaccine vehicle showed promising increases in overall survival [17, 18].

Another immunotherapeutic strategy, the blocking of CTLA-4 with ipilimumab led to enhanced T cell activation and increased tumor rejection [4, 19] and resulted in long-term tumor control in about 20 % of patients. As number of studies showed that BRAF inhibition increased the number of intratumoral cytotoxic T cells and increased expression of tumor-associated antigens on tumor cells [20–23], it was hypothesized that BRAF inhibition could synergize with CTLA-4 blockade. Unfortunately, the first trial to study combination of ipilimumab and vemurafenib was terminated prematurely due to severe hepatotoxicity [24]. This study showed that the combination of vemurafenib with checkpoint inhibition is not feasible, at least not when given simultaneously. Recently, other studies investigated the potential of combinations of more BRAF inhibitors, like dabrafenib, and checkpoint inhibition (ipilimumab) with or without additional treatment with the MEK inhibitor trametinib (NCT01940809 and NCT02130466). Preliminary results indicate that these combinations are tolerated, and do not result in severe hepatotoxicity. Nevertheless, the combination of three agents was discontinued based on the development of colitis followed by intestinal perforation [25–27]. We have recently shown that using natural blood-borne DC subsets as vehicles for DC vaccination, markedly improved responses in terms of overall survival could be observed, with minimal toxicity [17, 28]. With that in mind, we postulate that DC vaccination might be a candidate to be combined with vemurafenib. In this preclinical study, we investigated the feasibility of combining DC vaccination with vemurafenib by studying whether modulation of MAPK signaling pathway affected function and maturation of nDCs in healthy volunteers or melanoma patients receiving vemurafenib.

## Methods

### Cells

Isolation of nDCs was described previously [29]. Briefly, buffy coats were obtained from healthy volunteers with informed consent according to institutional and international guidelines. Blood from end-stage metastatic melanoma patients was collected prior to start of vemurafenib treatment and after 1 month vemurafenib therapy. This study was approved by the local Institutional Review Board (Committee on Research involving Human Subjects Arnhem-Nijmegen) and in accordance with the declaration of Helsinki. Written informed consent was obtained from all patients. After peripheral blood mononuclear cells (PBMC) isolation, plasmacytoid DCs (pDCs) were purified by positive isolation using anti-BDCA-4-conjugated magnetic microbeads, and BDCA-1^+^ myeloid DCs (mDCs) were purified using anti-CD1c-conjugated microbeads (both Miltenyi Biotec, Bergisch-Gladbach, Germany) after B cell depletion. Plasmacytoid DC and mDC purity was routinely up to 95 %, as assessed by double staining with BDCA-2/CD123 or CD11c/CD1c (all Miltenyi Biotec). DCs were cultured in X-VIVO-15 (Lonza, Verviers, Belgium) supplemented with 10 % human serum (Sanquin, Nijmegen, the Netherlands). DC maturation was induced through addition of 4 μg/ml R848 (Axxora, San Diego, CA). Monocyte-derived DCs (moDCs) were generated from adherent PBMCs, by culturing in the presence of IL-4 (500 U/ml) and GM-CSF (800 U/ml) (both Cellgenix, Freiburg, Germany). Cells were cultured in X-VIVO 15 medium supplemented with 2 % human serum and harvested on day 6 as immature DC. Immature DC or mDCs were activated through the addition of 4 μg/ml R848 and/or 20 μg/ml Poly I:C (Sigma-Aldrich).

### Flow cytometry

Purity of pDCs and mDCs after isolation and the phenotype of the pDC populations were determined by flow cytometry. The following primary monoclonal antibodies (mAbs) and the appropriate isotype controls were used: anti-BDCA1-FITC, BDCA2-PE, BDCA4-PE and CD123-APC (all Miltenyi Biotec); mIgG1-PE, mIgG1-APC, anti-CD11c-FITC or -APC, anti-HLA-ABC-PE (W6/32), anti-CD80-PE, or -APC, or -PeCy7, anti-CD86-PE, or -APC (all BD Bioscience Pharmingen, San Diego, CA, USA) anti-PD-L1-APC, anti-PD-L2-PE; anti-CD40-PE, anti-CD83-PE (Beckman Coulter, Mijdrecht, the Netherlands); anti-MHC-II-APC (eBioscience).

The phenotype of the DC populations after treatment with vemurafenib, dabrafenib, trametinib or a combination was determined by staining with the following antibodies and appropriate isotype controls: anti-CD80-PE-Cy7, anti-CD86 APC, anti-PD-L1-PE, anti-HLA-ABC-V450, anti HLA-DR-BV510, mIgG1-PE-Cy7, mIgG1-PE (all BD biosciences) and mIgG1-APC (eBioscience).

### Reagents

Vemurafenib (PLX4032 or RG7204) was kindly donated by Roche for study purpose. Vemurafenib was dissolved in DMSO to a stock concentration of 1 mg/ml. The vemurafenib stock was freshly diluted before each experiment to 60 μg/ml unless stated otherwise. Dabrafenib and trametinib were obtained from Alsachim (Illkirch-Graffenstaden, France). Both drugs were diluted freshly to 90 and 12 ng/ml, respectively.

### Antigen-specific T cell activation

pDCs and mDCs from a HLA-A2.1 + donor were loaded with different concentrations of a melanoma-specific peptide (gp100_280:288_) or irrelevant peptide (tyrosinase_369:376_) in 96-well round bottom plates (10 × 10^3^ cells per well). After approximately 2 h, R848, two concentrations of vemurafenib and gp100_280:288_-specific T cells (50 × 10^3^ cells per well) were added. After overnight incubation, CD69 and CD25 expression on the CD3^+^ gp100_280:288_-specific T cells was measured by flow cytometry using PE-Cy5 conjugated mouse anti-human CD69, APC-conjugated CD25 and BV421-conjugated CD3 (all BD biosciences).

### Mixed lymphocyte reaction

Allogeneic peripheral blood lymphocytes (PBLs) were co-cultured with differently matured pDCs, mDCs and moDCs in a 96-well round-bottom plate (pDC/PBL ratio 1:20 with 10^5^ PBLs). After 3 days of culture at 37 °C, 1 μCi/well ([0.037 MBq]/well; MP Biomedicals, Amsterdam, the Netherlands) of [^3^H]-thymidine was added for 12 h and incorporation was measured in a beta-counter.

### Cytokine detection

pDCs and mDCs were cultured overnight at a concentration of 10^5^ DCs/100 μl/well in a 96-well round-bottom (pDCs) or flat-bottom (mDC) plate. Supernatants were collected from DC cultures after 16 h of activation; IL-6, TNFα, RANTES, IP-10 and MIP-1α production was measured using a human Multiplex kit (BMS817FF from eBioscience) according to manufacturer’s instructions. IFNα production was measured by ELISA according to manufacturer’s instructions.

### Statistics

All experiments were performed at least three times and results are shown as the mean ± SEM. Data sets were either tested by a Student’s t test or by one-way ANOVA followed by Newman–Keuls or Dunnett’s multiple comparison test.

## Results

### At steady-state blood concentration vemurafenib inhibits nDC maturation and cytokine secretion in vitro

**Fig. 1:**
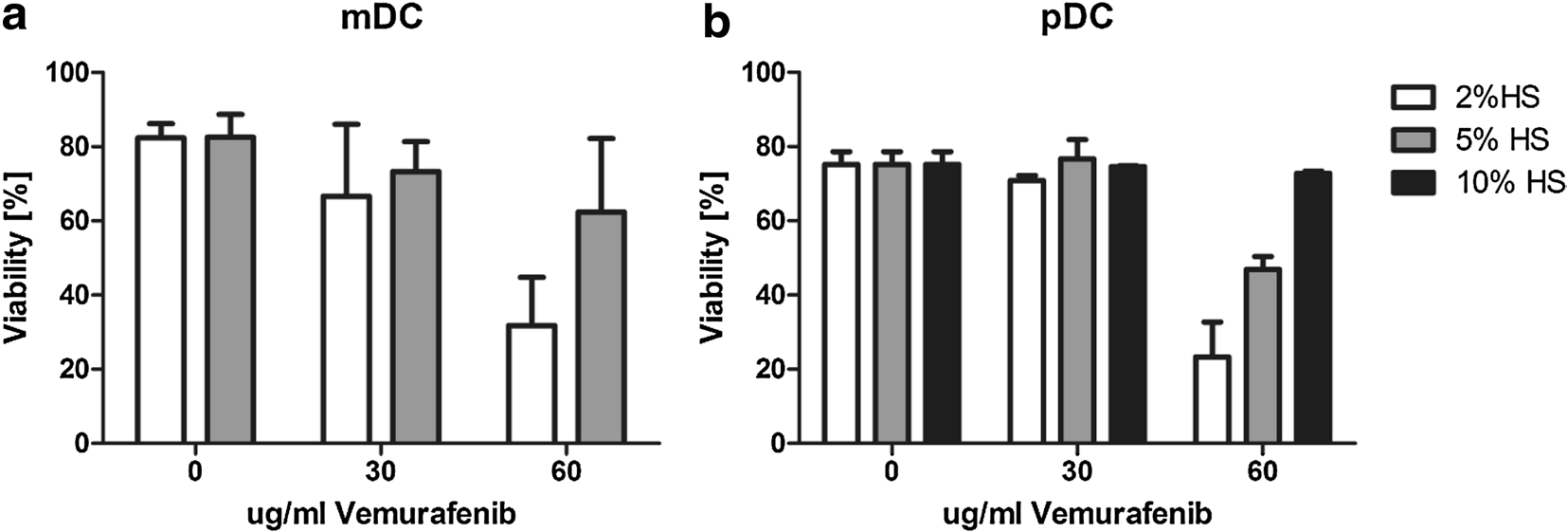
Vemurafenib bioavailability determines survival of pDCs and mDCs. Freshly isolated plasmacytoid DCs and myeloid DCs were cultured in increasing concentrations of vemurafenib and human serum and cell viability was determined after 24 h by FACS analysis

**Fig. 2:**
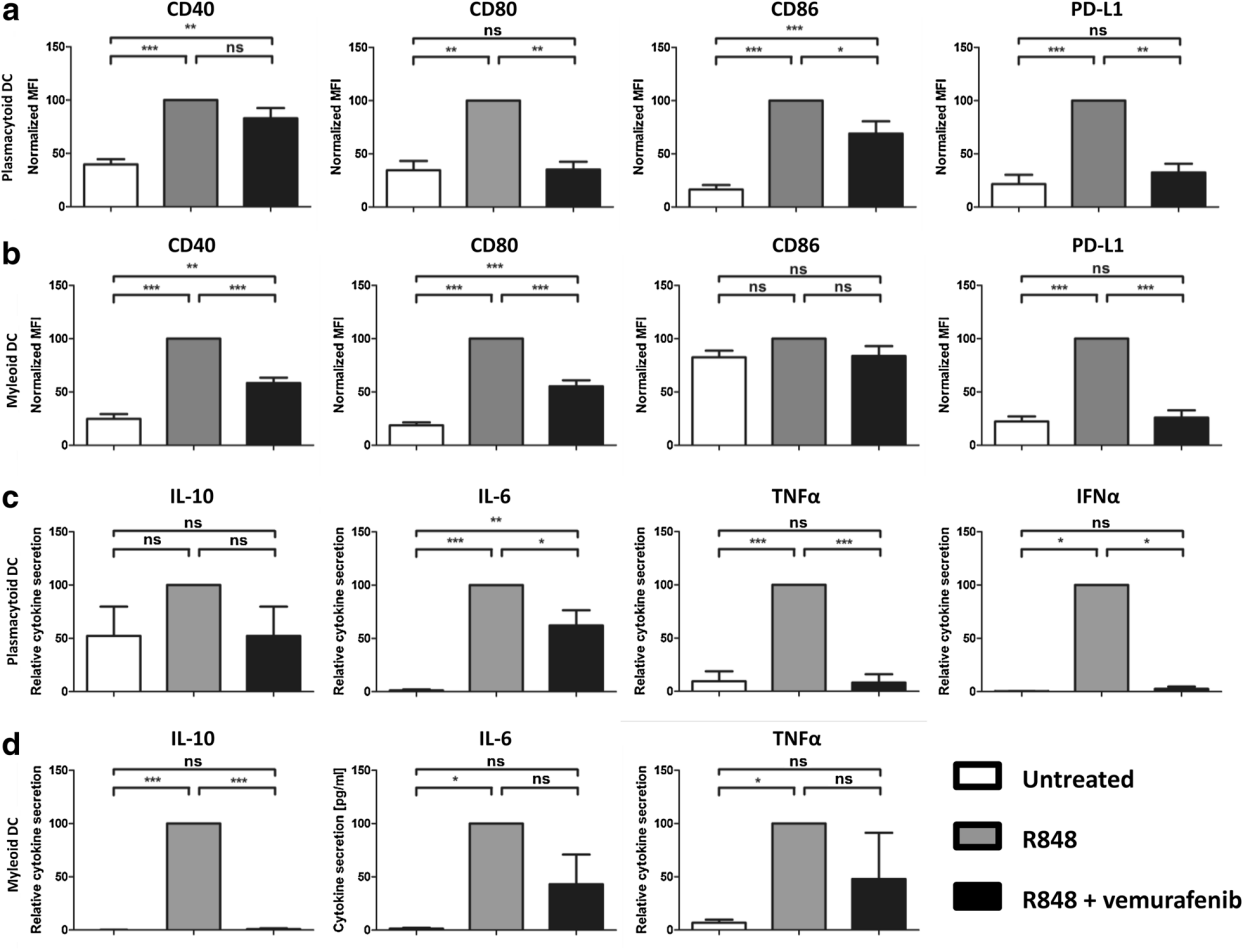
Vemurafenib impairs maturation of ex vivo cultured pDCs and mDCs. Freshly isolated pDCs (**a)** and mDCs (**b)** were cultured ex vivo and activated with R848 in presence or absence of 60 μg/ml vemurafenib. *Graphs* show the cell surface expression levels of CD80, CD86, CD40, and PD-L1 after 18 h. Freshly isolated pDCs (**c**) and mDCs (**d**) were cultured ex vivo and activated with R848 in presence or absence of 60 μg/ml vemurafenib. *Graphs* show the levels of the cytokines IL-10, IL-6, TNFα and IFNα (only pDCs) measured in the supernatant after 18 h. Shown is the mean (+SEM) of six independent experiments *P < 0.05, **P < 0.01,***P < 0.001

We explored whether vemurafenib had any effect on the viability, phenotype or maturation of the nDC subsets, pDCs and mDCs. Both DC subsets were exposed to 60 μg/ml of vemurafenib, which is higher than in most other studies and higher than the IC50 for most tumor cells in vitro [30]. However, 60 μg/ml is the average steady-state concentration (C_trough_; 34–93 μg/ml) of vemurafenib in the blood of patients treated with vemurafenib determined in pharmacokinetic studies [3, 31]. As such, this is the concentration of vemurafenib that circulating nDCs will be exposed to. This concentration of vemurafenib had deleterious effects on nDC viability cultured in medium containing 2 % human serum (Fig. 1; Additional file 1: Fig. S1). Taking into account that >99 % of vemurafenib in the blood is protein bound and the low amount of serum (and protein) used here, we investigated the effect of increasing the amount of serum to 5–10 %. DC viability was improved with both concentrations and 10 % serum restored pDC and mDC viability to the level of untreated cells (Fig. 1). In accordance with previous findings, vemurafenib had no negative effects on T cell and B cell viability (Additional file 2: Fig. S2) [20]. Additionally, there was no effect on the viability of pDCs and mDCs matured in the presence of vemurafenib at a concentration of 10 % human serum (Fig. 1). The next step was to investigate whether exposure to vemurafenib during maturation at a concentration of 10 % human serum had any effect on the resulting phenotype. The single stranded RNA analog R848 (agonist for TLR-7/8) was used to trigger DC maturation as it stimulates both mDCs and pDCs. R848 induces CD40, CD80 and CD86 indicative for maturation of both pDCs and mDCs (Fig. 2a, b). However, exposure to vemurafenib clearly hampered the maturation of these blood DC subsets as all markers failed to reach similar expression levels as cells cultured without vemurafenib.

Next, we determined whether vemurafenib could also modulate TLR-induced proinflammatory cytokine and chemokine secretion by pDCs and mDCs. We observed a marked decrease in the secretion of IL-6, TNF-a, and IP-10 by R848 stimulated pDCs and mDCs (Fig. 2c, d). Additionally, vemurafenib exposure also inhibited interferon-α (IFN-α) production by pDCs.

**Fig. 3:**
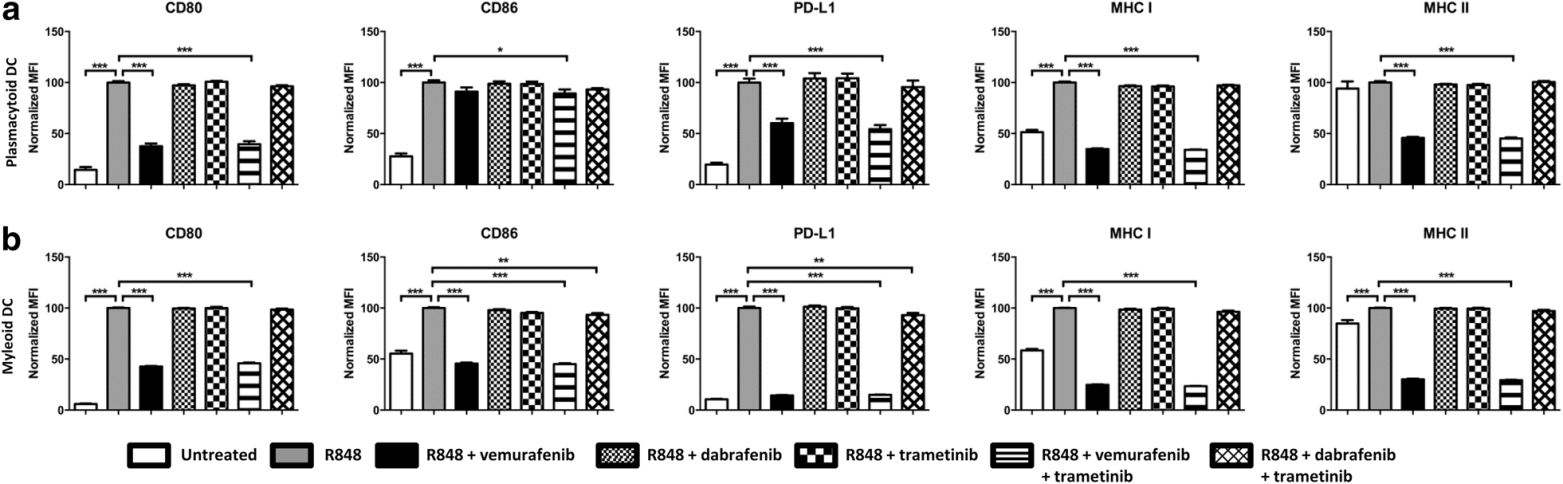
Dabrafenib and trametinib do not impair maturation of ex vivo cultured pDCs and mDCs. Freshly isolated pDCs (**a**) and mDCs (**b**) were cultured ex vivo and activated with R848 in presence or absence of 60 μg/ml vemurafenib, 90 ng/ml dabrafenib and 12 ng/ml trametinib. *Graphs* show the cell surface expression levels of CD80, CD86, PD-L1, MHC-I and MHC-II after 18 h. Shown is the mean (+SEM) of three independent experiments *P < 0.05, **P < 0.01,***P < 0.001

In order to investigate whether these observed effects were exclusively vemurafenib-related or class-specific for BRAF inhibition, we performed similar experiments with the BRAF inhibitor dabrafenib. Furthermore, we extended our analysis by studying the effects of the MEK inhibitor trametinib and the different combinations of these inhibitors. Importantly, the IC_50_ values for both dabrafenib and trametinib are approximately 40 times lower compared to vemurafenib under cell-free assay conditions. In vivo, this translates into much lower levels compared to vemurafenib, the C_through_ values for dabrafenib and trametinib are 90 and 12 ng/ml, respectively [32, 33], compared to 60 µg/ml for vemurafenib. We cultured mDCs and pDCs with the drugs either alone or in various combinations in the presence of R848. After overnight activation we observed that while vemurafenib inhibited the R848 induced-maturation of both pDCs and mDCs, neither dabrafenib nor trametinib had a significant impact on the upregulation of CD80, CD86, PD-L1, MHC-I or MHC-II (Fig. 3a, b). Similarly, the combination of vemurafenib and trametinib inhibited maturation just like vemurafenib while the combination of dabrafenib and trametinib did not (Fig. 3a, b). Taken together, vemurafenib exerts negative off-target effects on both mDCs and pDCs obtained from healthy donors, while other small molecule inhibitors with higher specificity and selectivity do not hinder mDC or pDC maturation in vitro.

### Exposure to vemurafenib leads to downregulation of MHC molecules, decreased allostimulatory capacity, and minimally decreased antigen-specific T cell activation

**Fig. 4:**
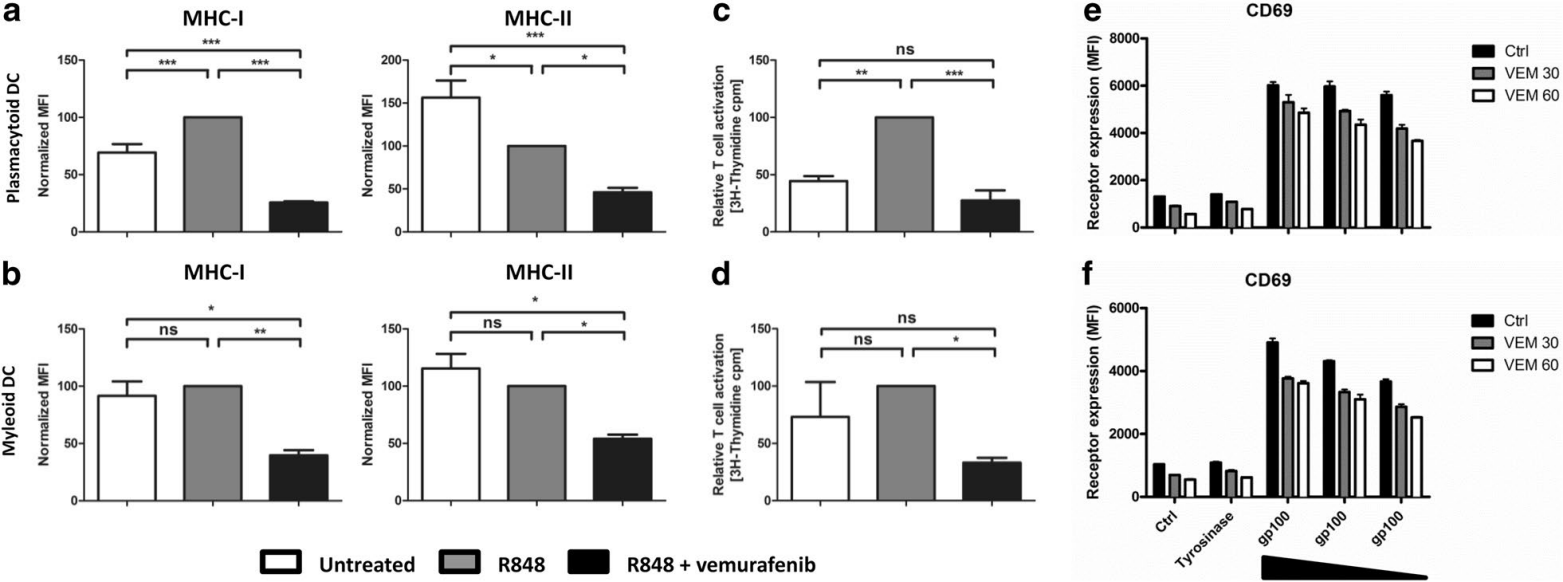
Vemurafenib impairs antigen presentation and allostimulatory capacity of ex vivo cultured pDCs and mDCs. Freshly isolated pDCs (**a**) and mDCs (**b**) were cultured ex vivo and activated with R848 in presence or absence of 60 μg/ml vemurafenib. Graphs show the cell surface expression levels of MHC class I and MHC class II after 18 h. Mixed lymphocyte reaction using freshly isolated, pDCs (**c**) and mDCs (**d**) stimulated with R848 in presence or absence of 60 μg/ml vemurafenib. After maturation DCs were incubated with allogeneic PBLs and T cell proliferation was measured after 4 days with [^3^H] thymidine incorporation. **d** pDCs and mDCs from a HLA-A2.1 + donor were loaded with different concentrations of a melanoma-specific peptide (gp100_280:288_) or irrelevant peptide (tyrosinase_369:376_) in the presence of R848 with/without vemurafenib and gp100_280:288_ specific T cells. Graphs show the cell surface expression levels of the early activation marker CD69 after overnight co-culture. Shown is the mean (+SEM) of at least three independent experiments *P < 0.05, **P < 0.01, ***P < 0.001

The expression levels of MHC class I, and MHC class II, which are necessary to activate CD8^+^ cytotoxic T cells, and CD4^+^ helper T cells, respectively was also measured. Although cytotoxic T cells are the most important mediators of anti-tumor immune responses, induction of CD4 T cells improves clinical outcome, probably by stimulating B cell function [34, 35]. In addition to decreased maturation of pDCs and mDCs, maturation-induced upregulation of MHC class I and MHC class II was also diminished after exposure to vemurafenib, indicating a possible decreased capacity of antigen presentation to T cells (Fig. 4a, b). Concomitantly, there was a significant decrease in allogeneic T cell proliferation induced by pDCs and mDCs matured in the presence of vemurafenib (Fig. 4c, d). The observed decrease in proliferation could not be due to increased expression of co-inhibitory molecules after DC stimulation in the presence of vemurafenib because PD-L2 was not expressed by either subset (data not shown) and PD-L1 upregulation was completely absent (Fig. 2a, b). Thus although vemurafenib inhibits the upregulation of PD-L1 the proliferation of allogeneic T cells is not enhanced but decreased, most likely due to the reduced expression of MHC molecules after stimulation of DC in the presence of vemurafenib. Furthermore, we examined whether vemurafenib affects the ability of pDCs and mDCs to prime antigen-specific T cells. For this we made use of Jurkat T cells that are transfected with the T cell receptor for gp100_280:288_. We observed that both pDCs and mDCs activated with R848 in the presence or absence of vemurafenib and loaded with various concentrations of the gp100_280:288_ peptide were able to significantly induce the activation of Jurkat T cells as evidenced by the specific upregulation of the IL-2 receptor CD25 (Additional file 3: Fig. S3) and the early activation marker CD69 (Fig. 4e, f). Although all conditions led to a significant activation compared to cells loaded with the irrelevant tyrosinase peptide, we observed that vemurafenib negatively affected the antigen-specific T cell receptor signaling as the level of CD69 induction was lower under this condition. Though a clear inhibitory effect on non-antigen specific T cell proliferation was observed, only a minute effect of vemurafenib on antigen-specific T cell activation could be detected. This discrepancy might be caused by prolonged (3 days) culture of T cells in the presence of vemurafenib, whereas in the antigen-specific T cell priming the readout was after 18 h. These findings reflect the potential in vitro toxicity of vemurafenib on immune cells. Taken together, these data imply that vemurafenib minimally decreased the antigen-specific immunostimulatory ability of blood DC subsets.

### The presence of immune cells mitigates the inhibitory effects of vemurafenib on DC maturation and cytokine secretion

**Fig. 5:**
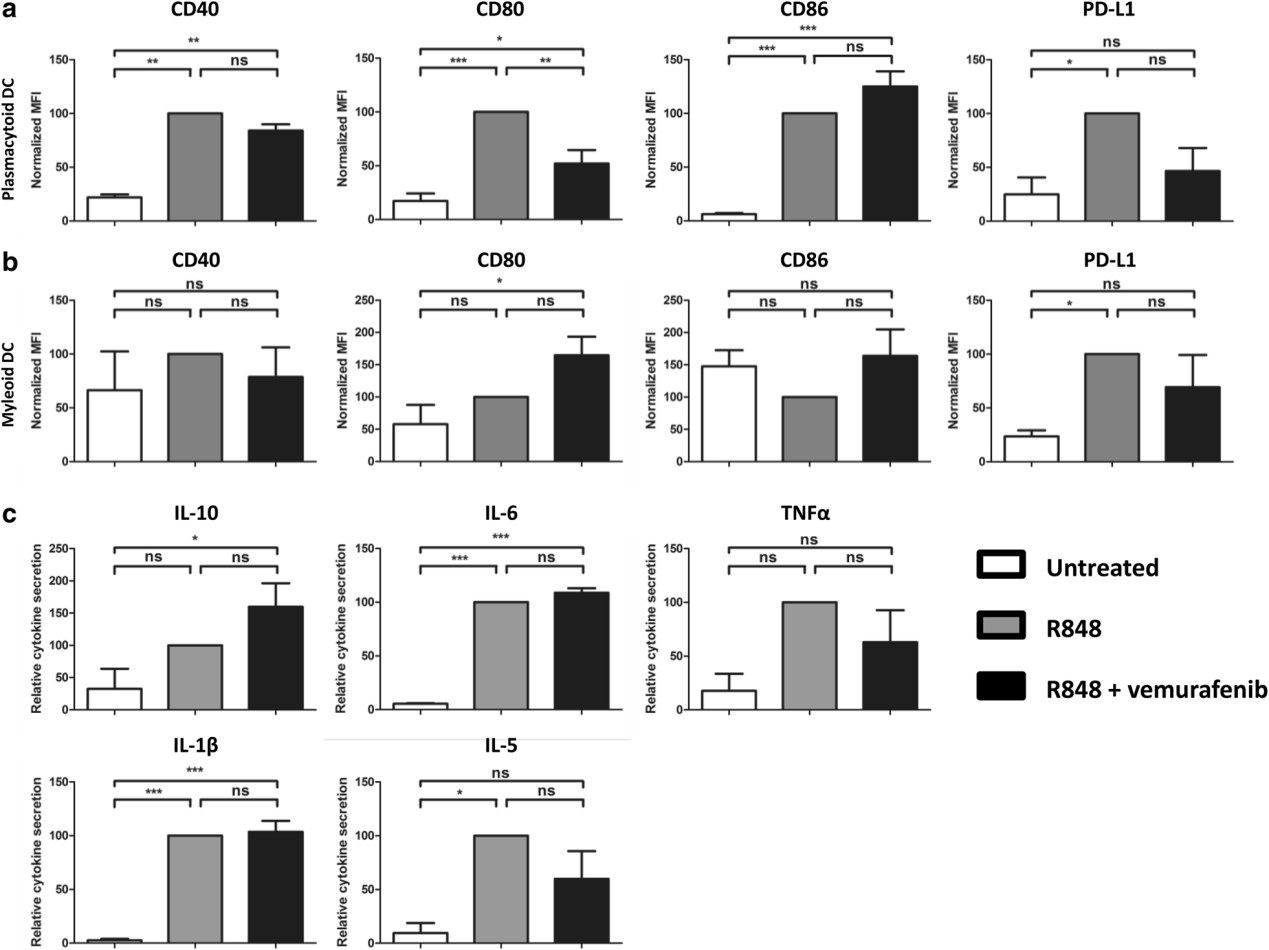
The presence of immune cells mitigates the inhibitory effects of vemurafenib on DC maturation and cytokine secretion. Freshly isolated PBMCs were cultured ex vivo and activated with R848 in presence or absence of 60 μg/ml vemurafenib. *Graphs* show the cell surface expression levels of CD80, CD86, CD40, and PD-L1 on pDCs (**a**) and mDCs (**b**) after 18 h. **c** *Graphs* show the levels of the cytokines IL-10, IL-6, TNFα, IL-1β, and IL-5 measured in the supernatant after 18 h. Shown is the mean (+SEM) of four independent experiments *P < 0.05, **P < 0.01, ***P < 0.001

Previous experiments studied the effects of vemurafenib on isolated, purified nDC cultures. We wondered whether the presence of other immune cells during the exposure to vemurafenib, as would be the case in the blood stream, would have an effect on the outcome. To test this, we stimulated whole PBMC fractions of healthy volunteers with R848 in the presence or absence of vemurafenib. After 24 h the PBMCs were harvested and stained for DC-specific markers and maturation markers. These strategies allowed us to gate the DCs and quantify the expression of maturation markers (Fig. 5). In contrast to the purified nDC cultures the inhibitory effect of vemurafenib on pDC maturation is less pronounced. There was a significant decrease in CD80 expression but there was no effect on CD86 expression (Fig. 5a). The maturation of mDCs on the other hand was hardly hampered by vemurafenib (Fig. 5b). PD-L1 upregulation is still inhibited by vemurafenib, showing that the compound is still active and reaching the cells. Furthermore, analysis of secreted cytokines in response to R848 with or without vemurafenib demonstrated no significant effects (Fig. 5c). Thus, vemurafenib has little to no deleterious effects in total PBMC cultures.

### Vemurafenib does not inhibit ex vivo maturation of pDCs and mDCs isolated from melanoma patients on active treatment

**Fig. 6:**
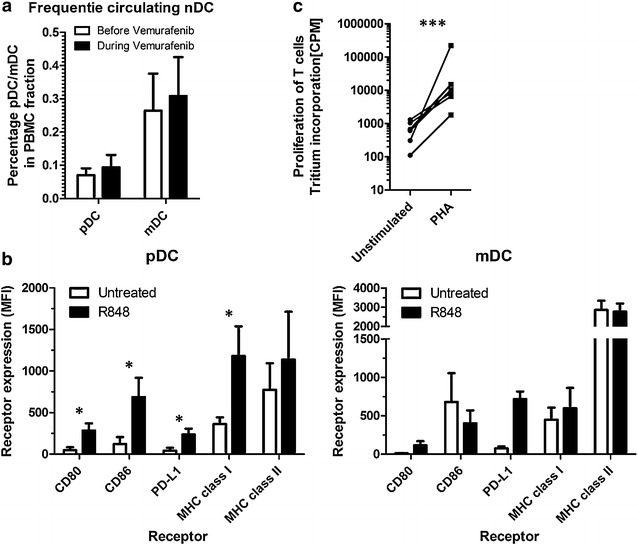
Vemurafenib does not inhibit ex vivo maturation of pDCs and mDCs isolated from melanoma patients on active treatment. PBMCs were isolated from patients before and after 1 month on vemurafenib treatment. **a** The frequency of pDCs and mDCs in the PBMCs was measured by FACS analysis. **b** Freshly isolated PBMCs from melanoma patients after 1 month on vemurafenib treatment were cultured ex vivo and activated with R848. *Graphs* show the cell surface expression levels of CD80, CD86, PD-L1, MHC-I, and MHC-II on pDCs and mDCs after 18 h. **c** Freshly isolated PBMCs from melanoma patients after 1 month on vemurafenib treatment were stimulated with PHA and T cell proliferation was measured after 4 days with [^3^H] thymidine incorporation. Shown is the mean (+SEM) of seven independent experiments *P < 0.05, **P < 0.01, ***P < 0.001

We isolated PBMCs from three melanoma patients before and after 1 month receiving vemurafenib 960 mg BID. We did not observe any significant change in the frequency of either pDCs or mDC after one month of vemurafenib treatment (Fig. 6a). We investigated the effect of vemurafenib on pDC and mDC maturation by stimulating freshly isolated PBMCs from patients on vemurafenib treatment in vitro with R848. These pDCs and mDCs matured normally, indicated by the significant increase in CD80, CD86, PD-L1 and MHC-I (only for pDCs) (Fig. 6b). Additionally, the levels of DC maturation before start of vemurafenib and after one month on vemurafenib were similar (Additional file 4: Fig. S4). Next we investigated the effect of vemurafenib treatment on T cell activation. We isolated PBMCs from seven Patients receiving vemurafenib and induced non-specific T cell proliferation by addition of PHA, which led to significant T cell proliferation in all cases (Fig. 6c). From a subset of these patients we also compared T cell proliferation before and 1 month after start of vemurafenib and saw no differences (Additional file 4: Fig. S4) From these data, we can conclude that vemurafenib does not negatively affect the frequency of nDC subsets in the blood, nor affects their maturation and also has no negative effects on T cell function.

## Discussion

Approximately 50 % of melanoma tumors have an activating mutation in BRAF V600 [8, 9]. This has led to the development of specific BRAF inhibitors such as vemurafenib and dabrafenib. Despite the rapid and impressive response rates, treatment with vemurafenib is limited due to the short duration of the response caused by rapid onset of resistance [2, 12, 36]. Other therapeutic opportunities in order to improve the efficacy of vemurafenib might be found in the combination with DC vaccination. Currently, DC vaccination faces the opposite set of challenges compared to vemurafenib. Many patients do not respond to initial treatment, possibly due to the enormous challenge of fighting a high tumor load in late stage patients. However, the patients that do respond have long-lasting tumor control with life expectancy measured in years instead of months [17, 34, 37, 38]. This provides the tantalizing possibility of combining vemurafenib with DC vaccination. Vemurafenib treatment could lead to a rapid decrease in tumor load, giving the immune system enough time to mount an effective anti-tumor response that can eliminate residual tumor cells and vemurafenib resistant tumor cells.

Here, we performed an exploratory preclinical, ex vivo, study on the feasibility of combining vemurafenib treatment with DC vaccination; specifically, we studied the potential off-target effects of vemurafenib on human natural blood DC subsets, which appear to be the superior vehicles for DC vaccination [17, 28, 39]. We found that the availability of vemurafenib is a crucial component in testing its toxicity. In this study, we used the concentration of vemurafenib, which is found at steady state pharmacokinetics in patients undergoing treatment [31, 40]. This is in contrast to other studies that have looked at the toxicity of vemurafenib on immune cells, including lymphocytes and monocyte-derived DCs using very low concentrations of vemurafenib (0.5–5 µg/ml) [20, 41]. We reasoned that our chosen concentration was the amount of vemurafenib that these blood-borne DCs would be exposed to. Initial experiments showed that this concentration of 60 µg/ml was toxic for pDCs and mDCs. Increased levels of human serum relieved this toxicity, which could be attributed to the fact that the majority of vemurafenib in the blood stream (>99 %) is protein bound, thus reducing the concentration of free vemurafenib [42]. These results already illustrate that it is very difficult to recreate the correct physiological contexture when doing in vitro experiments and that one should always exercise caution when drawing conclusions. So at this concentration vemurafenib had no effect on nDC viability, but we did observe a clear and significant inhibition of DC maturation and cytokine secretion. However, when replicating this experiment using the whole PBMC fraction instead of the purified nDC subsets, we observed that vemurafenib had no effect on DC maturation, or cytokine secretion by the PBMCs. This could again be attributed to vemurafenib availability and thus the limited uptake of vemurafenib by the pDCs and mDCs. Finally, we studied pDCs and mDCs in PBMC fraction obtained from patients before and after 1 month of vemurafenib treatment. Likewise, DCs were not hampered in their ability to mature. Taken together our results clearly show no sign of negative effects of vemurafenib on DC subsets of patients undergoing treatment. DC frequencies remain the same during treatment and also their function and maturation is unaffected. In all, the combination of vemurafenib with DC vaccination seems feasible.

One major concern with combination regimens is the increased risk of toxicity. Indeed the combination of vemurafenib with ipilimumab was unsuccessful because of increased hepatotoxicity. Currently, a phase II study evaluating the safety and benefit of sequential treatment with vemurafenib and ipilimumab is ongoing (NCT01673854) which is an approach to potentially decrease toxicity while gaining activity. The PD-1 receptor is another inhibitory receptor on T-cells that functions as an immune checkpoint [43, 44]. Monoclonal antibodies that block PD-1, or its ligand PD-L1 (which is expressed on tumor cells) have demonstrated excellent clinical activity in patients with metastatic melanoma, while generally being less toxic than ipilimumab [5–7, 45]. One multi-modality approach would be the combination of vemurafenib with PD-1 or PD-L1 blockade. The combination vemurafenib and PD-L1 blockade is currently under investigation (NCT01656642). In light of our results, we would propose the combination of vemurafenib with DC vaccination. An increase in toxicity due to this combination treatment is not foreseen, as the toxicity level of DC vaccination on its own, is low consisting mostly of flu-like symptoms (Grade 1–2 toxicity) [16, 17, 46, 47]. Other interesting targeted therapies are dabrafenib, trametinib, or the combination of dabrafenib and trametinib and combining those with DC vaccination. Indeed the combination of dabrafenib and trametinib seems to be more effective while not having a higher toxicity profile than vemurafenib monotherapy [27], though for the moment vemurafenib is still first line therapeutic option.

Lastly, in biopsies of melanoma metastases, an increased T cell infiltration after vemurafenib treatment was seen. Furthermore, a correlation between CD8 infiltration and clinical response was observed [22]. This again indicates no negative effects of vemurafenib on tumor-specific immune responses. Indeed the increased expression of tumor associated antigens by tumor cells after exposure to vemurafenib might even synergize with the activation of tumor-specific T cells by the DC vaccine [23], making this combination all the more attractive.

## Conclusion

Our data shows that vemurafenib does not inhibit the functionality of naturally circulating DC subsets in the context of a complete immune system. Additionally, we show that pDCs and mDCs isolated from metastatic melanoma patients treated with vemurafenib display normal functionality. Given the extremely mild toxicity profile of DC vaccination, our results pave the way for a combinatorial treatment strategy consisting of combining vemurafenib with pDC and/or mDC vaccination. This protocol would combine the best of both worlds, rapid and efficient tumor debulking by vemurafenib with long lasting anti-tumor immune responses induced by DC vaccination.

## Additional files

10.1186/s12967-016-0844-6 Freshly isolated pDCs were cultured ex vivo and activated with R848 in presence or absence of increasing concentration of vemurafenib. Graphs show the levels IFNα measured in the supernatant after 18 h. Shown is the mean (+SEM) of three independent experiments.
10.1186/s12967-016-0844-6 Indicated immune cell subsets were cultured in increasing concentrations of vemurafenib and cell viability was determined after 24 h by FACS analysis. Shown is the mean (+SEM) of three independent experiments.
10.1186/s12967-016-0844-6 Vemurafenib has minimal negative effect on gp100-specific T cell priming. pDCs and mDCs from a HLA-A2.1 + donor were loaded with different concentrations of a melanoma-specific peptide (gp100_280:288_) or irrelevant peptide (tyrosinase_369:376_) in the presence of R848 with/without vemurafenib and gp100_280:288_ specific T cells. Graphs show the cell surface expression levels of CD25 after overnight co-culture. Shown is the mean (+SEM) of three independent experiments.
10.1186/s12967-016-0844-6 (A) PHA induced T cell proliferation before and after 1 month on vemurafenib treatment. (B) Freshly isolated PBMCs from melanoma patients before and after one month on vemurafenib treatment were cultured ex vivo and activated with R848. Graphs show the cell surface expression levels of CD80, CD86, PD-L1, MHC-I, and MHC-II on pDCs and mDCs after 18 h.
