# Supplementary figures and images for: Preclinical exploration of combining plasmacytoid and myeloid dendritic cell vaccination with BRAF inhibition
Source: J Transl Med. 2016 Apr 14;14:88. doi: 10.1186/s12967-016-0844-6 (PMC4831164; doi:10.1186/s12967-016-0844-6)

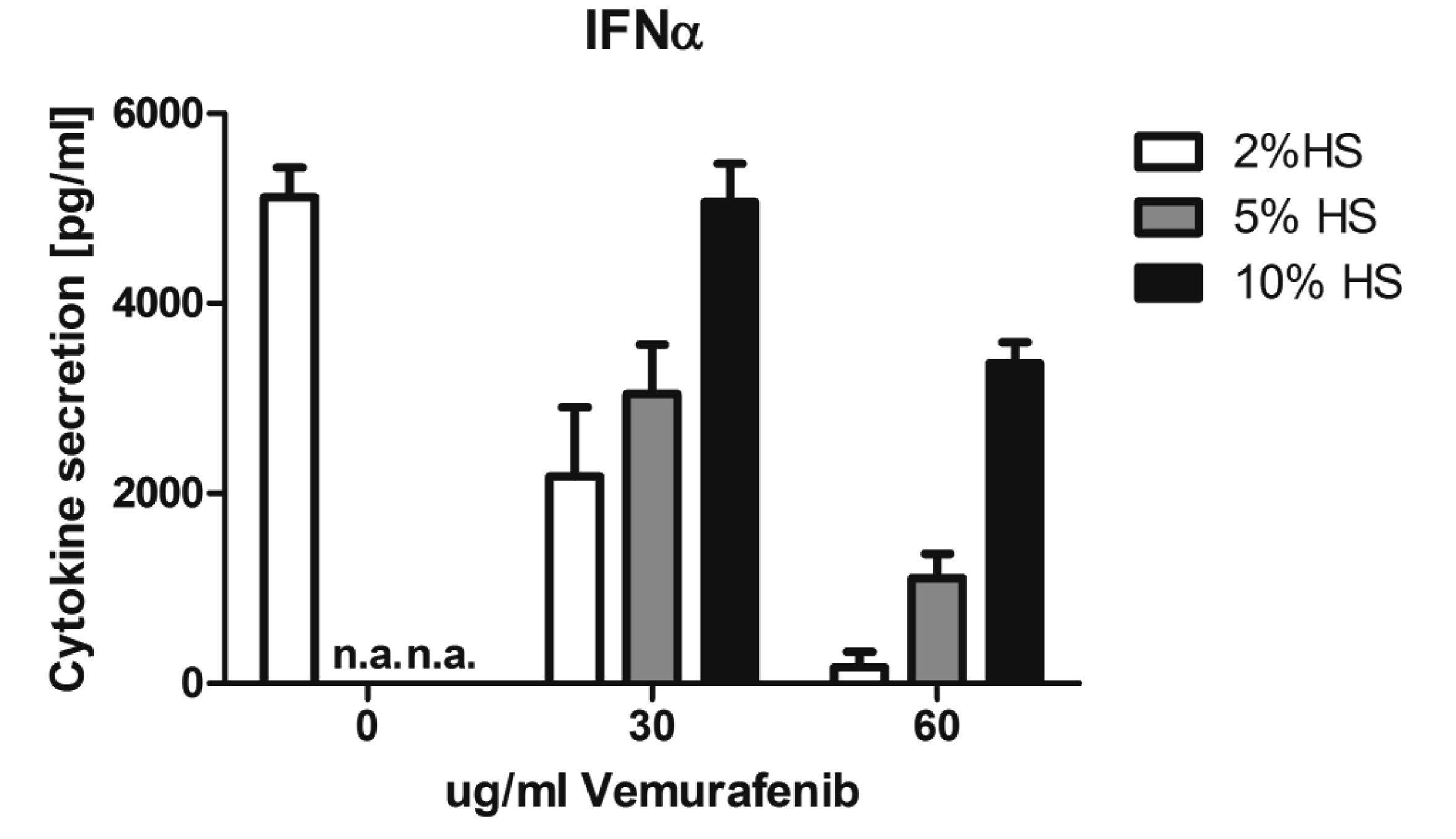

Supplement: Supplementary file 1 — 10.1186/s12967-016-0844-6 Freshly isolated pDCs were cultured ex vivo and activated with R848 in presence or absence of increasing concentration of vemurafenib. Graphs show the levels IFNα measured in the supernatant after 18 h. Shown is the mean (+SEM) of three independent experiments. [file 12967_2016_844_MOESM1_ESM.tif]

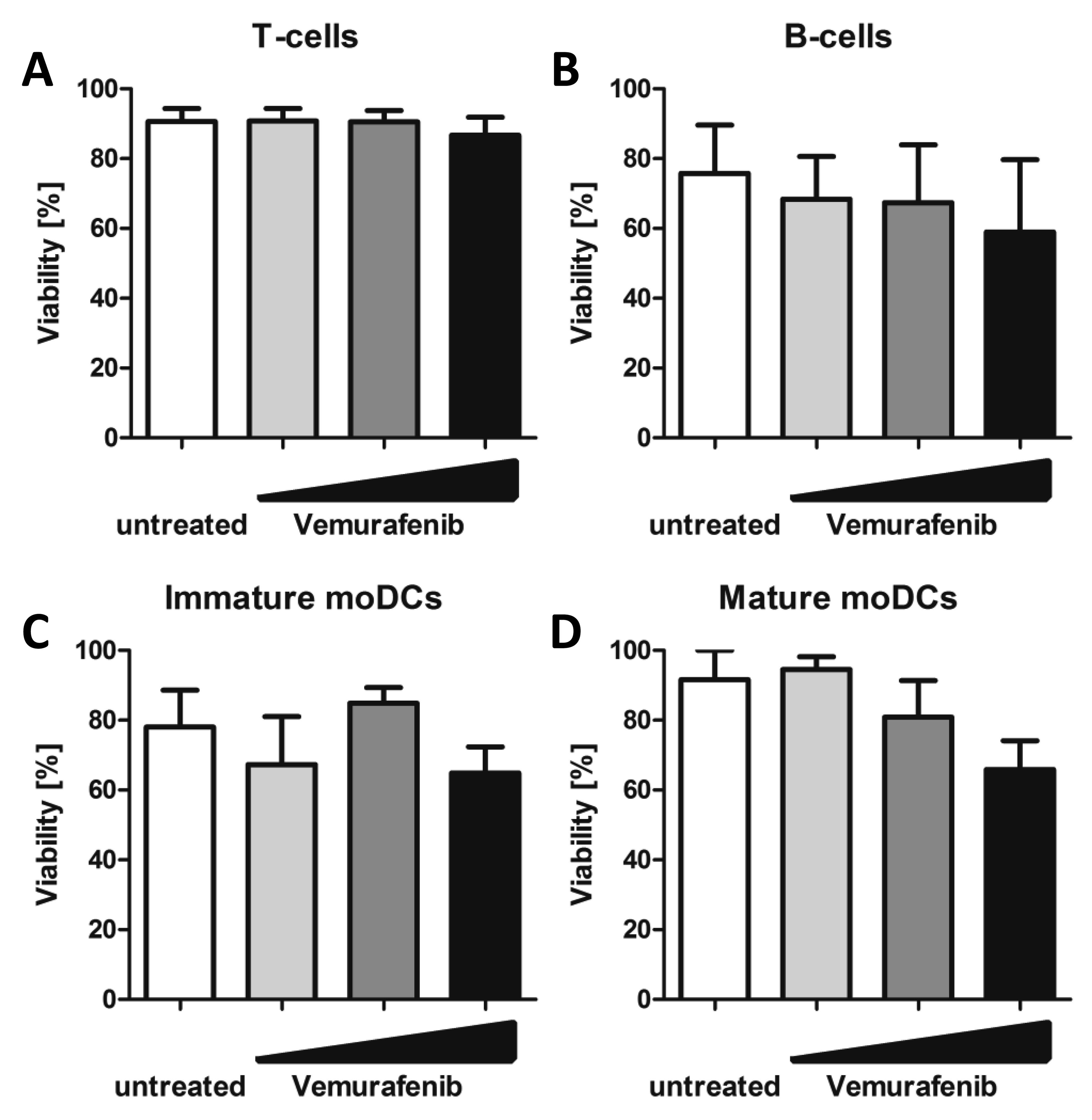

Supplement: Supplementary file 2 — 10.1186/s12967-016-0844-6 Indicated immune cell subsets were cultured in increasing concentrations of vemurafenib and cell viability was determined after 24 h by FACS analysis. Shown is the mean (+SEM) of three independent experiments. [file 12967_2016_844_MOESM2_ESM.tif]

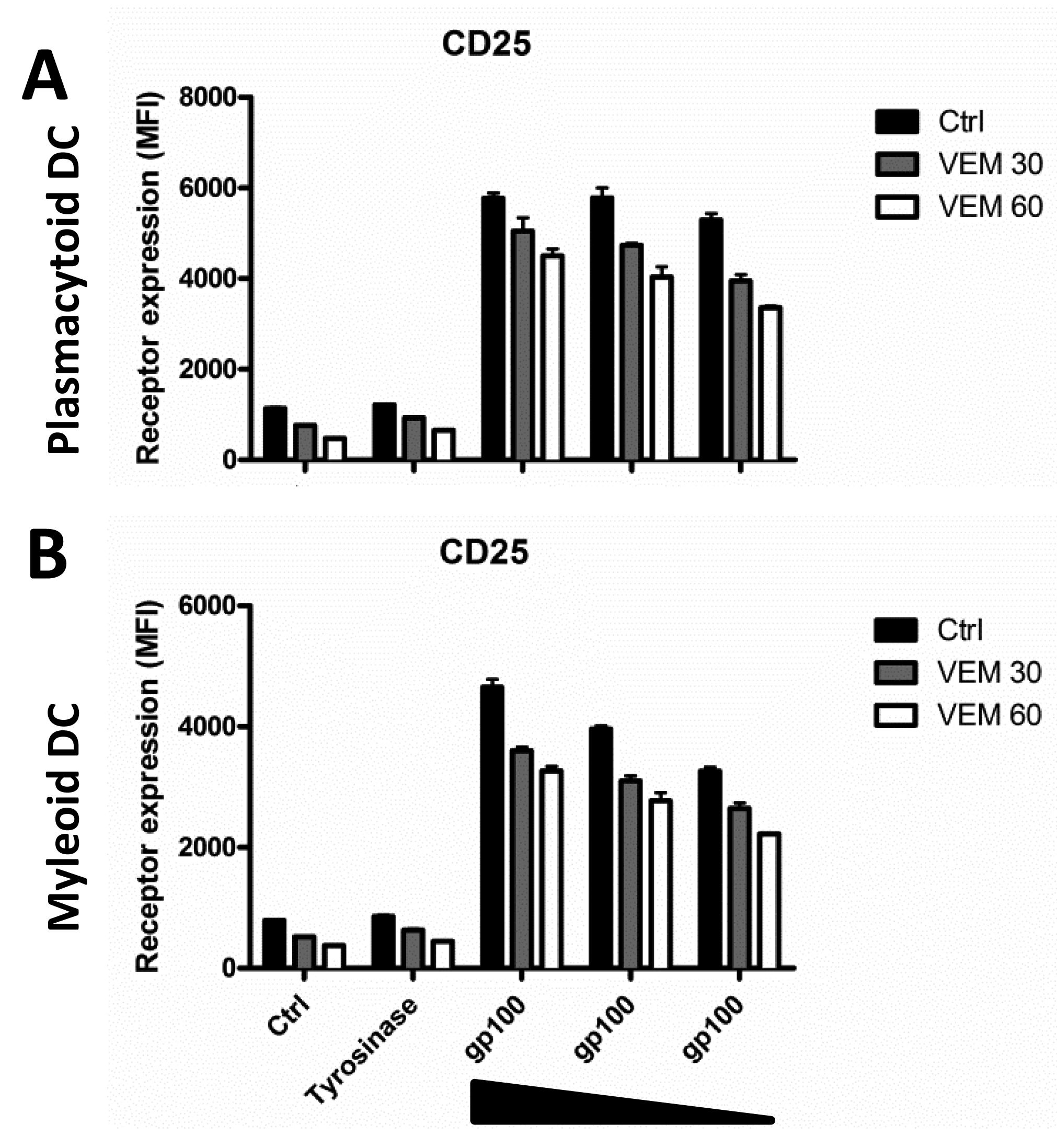

Supplement: Supplementary file 3 — 10.1186/s12967-016-0844-6 Vemurafenib has minimal negative effect on gp100-specific T cell priming. pDCs and mDCs from a HLA-A2.1 + donor were loaded with different concentrations of a melanoma-specific peptide (gp100280:288) or irrelevant peptide (tyrosinase369:376) in the presence of R848 with/without vemurafenib and gp100280:288 specific T cells. Graphs show the cell surface expression levels of CD25 after overnight co-culture. Shown is the mean (+SEM) of three independent experiments. [file 12967_2016_844_MOESM3_ESM.tif]

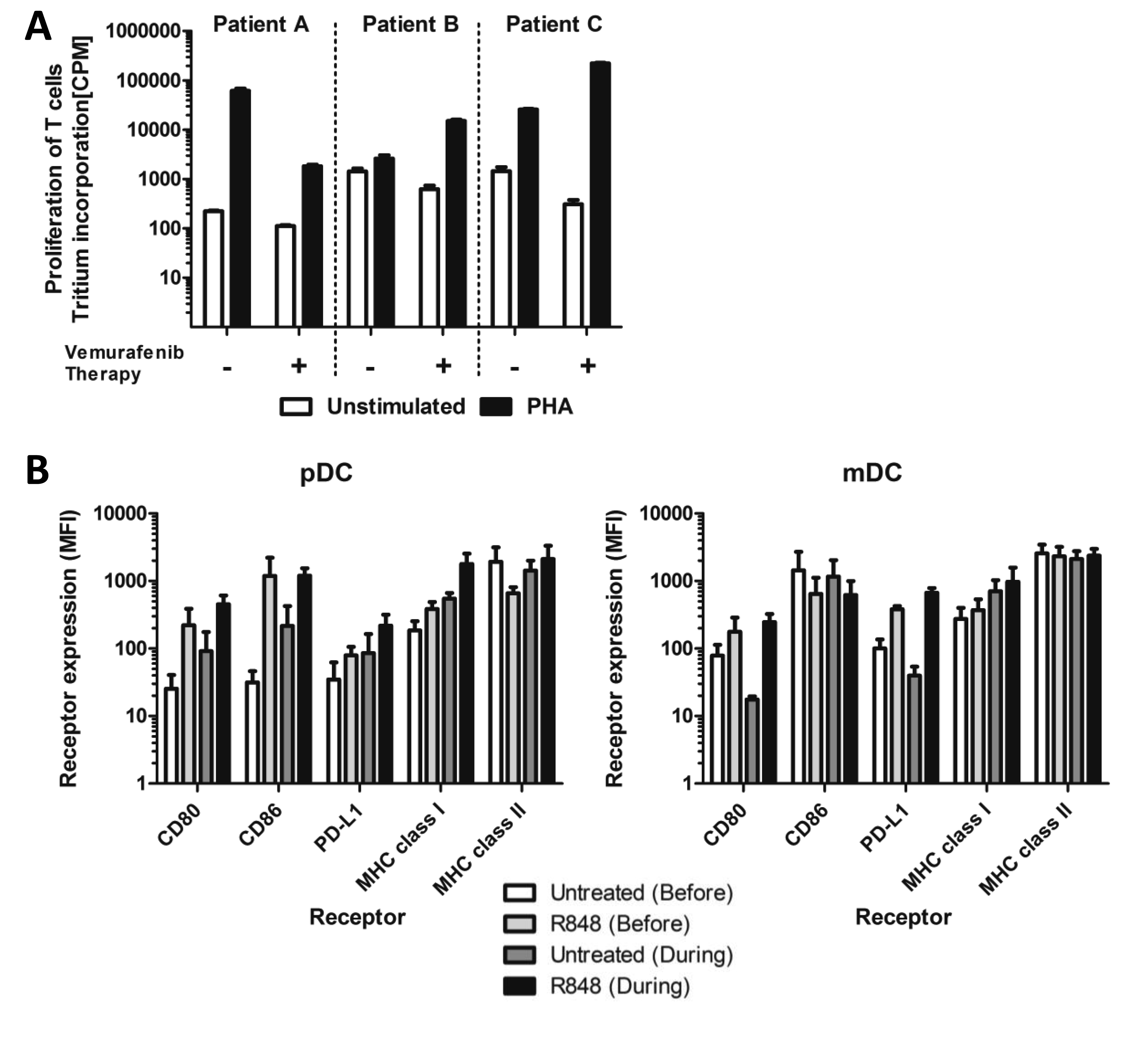

Supplement: Supplementary file 4 — 10.1186/s12967-016-0844-6 (A) PHA induced T cell proliferation before and after 1 month on vemurafenib treatment. (B) Freshly isolated PBMCs from melanoma patients before and after one month on vemurafenib treatment were cultured ex vivo and activated with R848. Graphs show the cell surface expression levels of CD80, CD86, PD-L1, MHC-I, and MHC-II on pDCs and mDCs after 18 h. [file 12967_2016_844_MOESM4_ESM.tif]
